# Therapeutic strategies for hypertension: exploring the role of microbiota-derived short-chain fatty acids in kidney physiology and development

**DOI:** 10.1007/s00467-025-06883-2

**Published:** 2025-07-10

**Authors:** Giovane G. Tortelote

**Affiliations:** Section of Pediatric Nephrology, Department of Pediatrics, Tulane University School of Medicine, New Orleans, LA 70112 USA

**Keywords:** Kidney, Gut microbiota, Short-chain fatty acids, SCFA, Kidney development, Chronic kidney disease, CKD, Hypertension, G-protein coupled receptors, Nutrition

## Abstract

Gut microbiota have emerged as a key regulator of systemic health, influencing various physiological processes, including kidney development, function, and blood pressure regulation. This review highlights the role of microbiota-derived short-chain fatty acids (SCFAs), primarily acetate, propionate, and butyrate, in the gut-kidney axis, focusing on their signaling mechanisms, vascular effects, and developmental implications. Evidence suggests that SCFAs modulate kidney development and function and exert anti-inflammatory, antioxidant, and vasoregulatory effects through specific G protein-coupled receptors (GPR41, GPR43, GPR109A, OLFR78, and OLFR558). Human studies and research using genetically modified animals have demonstrated that gut dysbiosis disrupts SCFA metabolism, potentially contributing to hypertension, endothelial dysfunction, and chronic kidney disease (CKD). Germ-free microbiota-transplantation studies revealed that the presence of gut microbiota directly influences vascular tone and systemic blood pressure via SCFA-mediated mechanisms. Furthermore, acetate, a SCFA, is shown to impact fetal kidney development and nephron progenitor cell dynamics. Sex-specific effects of gut microbiota on vascular remodeling and immune responses further highlight the complexity of microbiome-host interactions. In pediatric patients, altered SCFA profiles are associated with CKD progression and relapse in nephrotic syndrome. Clinical data suggest that plasma SCFA levels may serve as biomarkers for hypertension risk and cardiovascular outcomes in children with kidney disease. Therapeutically, interventions targeting SCFA pathways, such as probiotics, prebiotics, dietary fiber diet, and receptor agonists, may help restore gut-kidney axis balance and improve kidney and cardiovascular outcomes. This review illustrates the critical role of SCFAs as mediators linking the gut microbiota to kidney and vascular health. Continued investigation into SCFA signaling may uncover novel strategies for preventing and managing hypertension, CKD, and developmental nephropathies.

**Graphical Abstract:**

**Figure:**
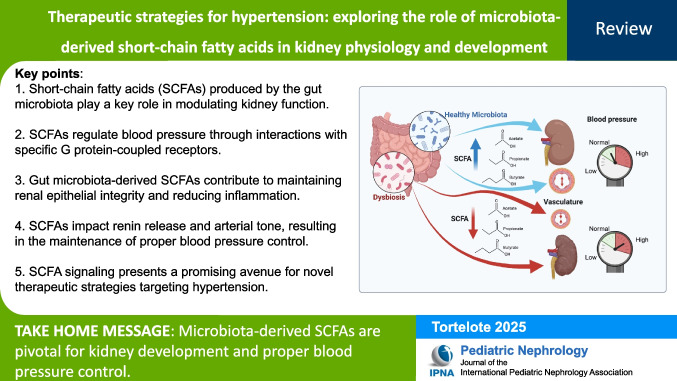
A higher resolution version of the Graphical abstract is available as Supplementary information

**Supplementary Information:**

The online version contains supplementary material available at 10.1007/s00467-025-06883-2.

## The importance of gut microbiota to host physiology and blood pressure regulation

The mutualistic relationship between gut microbiota and human health is undeniably an evolutionary triumph. The human gastrointestinal tract hosts a complex and ever-changing community of microorganisms known as the gut microbiota. This diverse ecosystem comprises trillions of microbes, including bacteria, archaea, viruses, and fungi [1]. The gut microbiota favor the host by supporting digestion, synthesizing essential vitamins, modulating immune responses, metabolism, cell signaling, and epigenetic regulation. In return, the host supplies nutrients and a stable environment, fostering a symbiotic relationship [2]. This mutually beneficial interaction is best described as mutualistic, rather than merely commensal.

The importance of the microbiota in host health and disease is well recognized today. A growing body of evidence now supports the wide-reaching influence of the gut microbiota on distant organs, including the kidneys [2–4]. This modern understanding resonates with the ancient saying often attributed to Hippocrates,"All diseases begin in the gut", a statement that, while not scientifically accurate in its original context, underscores the central role of the gut in systemic health.

The metabolites produced by the gut microbiota are the main route of communication with the host and play several physiological and pathological roles. Several metabolites produced by the gut microbiota have been identified as modulators of host physiology, including trimethylamine N-oxide (TMAO), ketone bodies, aromatic amino acids, and shikimic acid, among many others [4–11]. Short-chain fatty acids (SCFAs) stand out as pivotal gut microbiota-derived metabolites mediating host-microbe communication. Recent efforts suggest that SCFA signaling contributes to kidney development and function, thereby presenting a new therapeutic avenue in the context of the so-called gut-kidney axis and blood pressure regulation [3, 12].

**Fig. 1:**
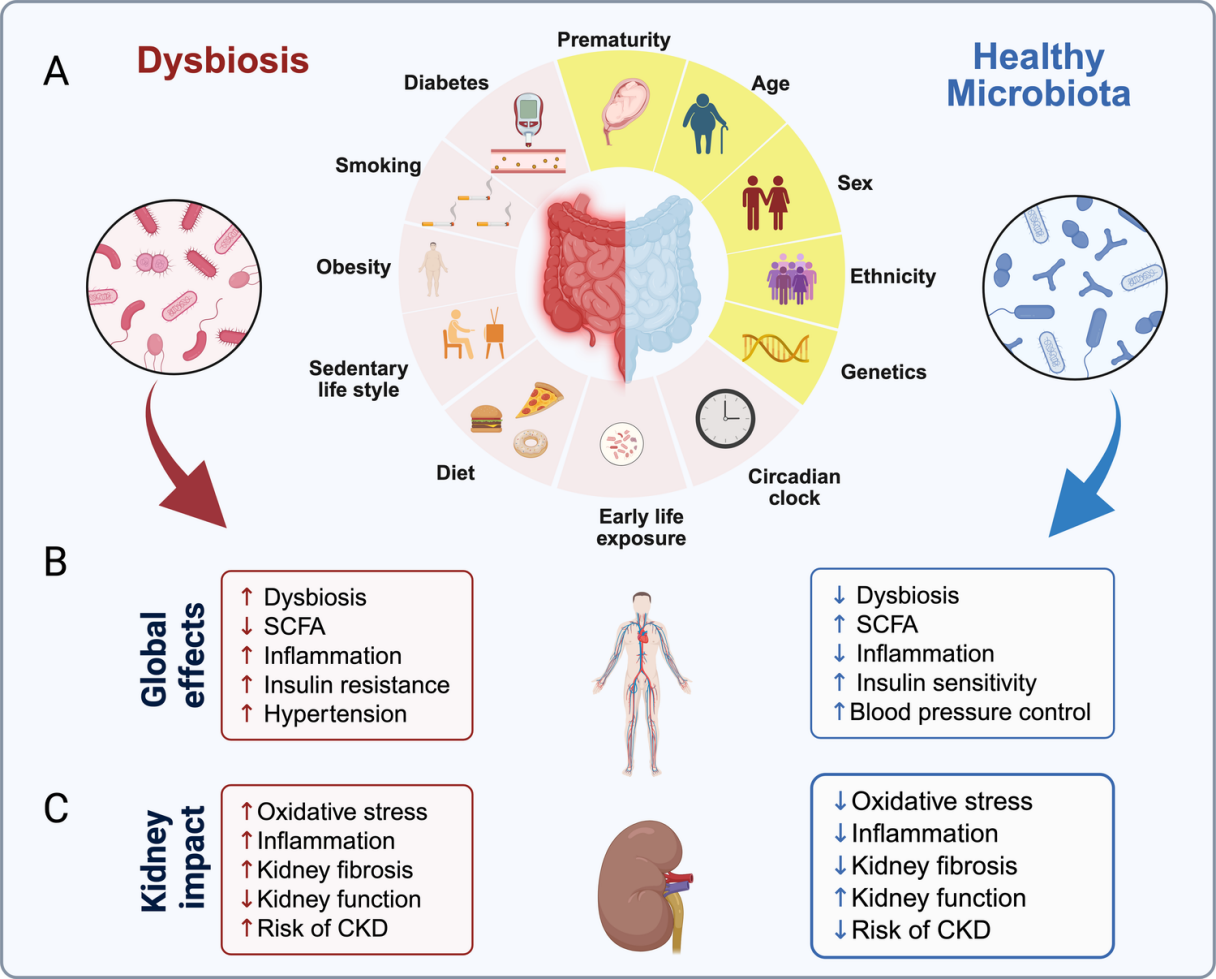
Impact of gut microbiota composition on systemic and kidney health. (**A**) Various non-modifiable (yellow) and modifiable (pink) factors influence the gut microbiota composition throughout life. Non-modifiable variables such as genetics, ethnicity, sex, and age can impact microbiota formation regardless of host actions. On the other hand, modifiable variables such as diet, sedentary lifestyle, obesity, smoking, diabetes, and early-life exposures are known to promote gut dysbiosis. (**B**) Globally, gut dysbiosis is associated with decreased SCFA production, increased systemic inflammation, insulin resistance, and hypertension. In contrast, a healthy microbiota increases SCFA levels, reduces inflammation, improves insulin sensitivity, and supports adequate blood pressure regulation. (**C**) Dysbiosis contributes to kidney damage through increased oxidative stress, inflammation, and fibrosis, leading to impaired kidney function and increased CKD risk. Conversely, a healthy microbiota reduces these deleterious processes, preserving kidney function and lowering CKD risk

An individual's microbiota composition is typically unique, shaped by a combination of modifiable and non-modifiable factors (Fig. 1A). Non-modifiable factors, such as genetics, age, premature birth, and sex, shape the initial microbiota composition. However, environmental (modifiable) factors such as geography, diet, lifestyle, and early-life microbial exposures play a decisive role in determining the final composition of the gut microbiota [13–16].

Changes imposed by modern society, such as circadian rhythm alterations (e.g., work shift), Western diet, use of antibiotics, and sedentary lifestyle, also impact the gut microbiota [17, 18]. These changes are known to favor microbial imbalances and the development of dysbiosis that correlates with several pathological conditions, including metabolic disease [19], inflammation [20], oxidative stress and cancers [4], hypertension [21], and chronic kidney disease (CKD) [22, 23]. Figures 1B and C illustrate some of the impacts of a healthy microbiota vs. dysbiosis on the host’s physiology.

Notably, the gut microbiota appears to play a role in CKD development. Briskey et al. [24] reported that dysbiosis promoted the accumulation of uremic toxins, exacerbating disease progression in CKD patients. Thus, could a healthy microbiota benefit kidney function and blood pressure regulation? The answer is yes; recent literature has indicated that a healthy gut microbiota and their products, SCFAs, correlate with kidney health and adequate blood pressure regulation, discussed below.

## Gut microbiota and blood pressure regulation

Initial evidence suggesting a connection between gut microbiota imbalances and hypertension started in the middle of the last decade [25, 26]. Recent experiments with fecal transplantation indicated that the relationship between gut microbiota and hypertension is causal rather than merely correlative. One key study by Li et al. [27] showed that transplanting microbiota from hypertensive individuals into germ-free mice induced a hypertensive phenotype, directly implicating the gut microbiota in blood pressure regulation. Similarly, Wilck et al. [28] demonstrated that exposure to a high-salt diet disrupted the gut microbiome and depleted the *Lactobacillus* phylum, in mice. Interestingly, these changes were associated with increased Th17 cell activity, which exacerbated both inflammation and salt-sensitive hypertension. Reintroducing beneficial bacteria prevented these effects by restoring Th17 balance. Notably, a moderate high-salt diet in humans also reduced *Lactobacillus* abundance, increased Th17 cells, and elevated blood pressure. These findings indicate that fostering a healthy gut microbiota could mitigate salt-sensitive hypertension via modulation of the gut-kidney-immune axis.

Of note, the discovery of Th17 cells as a distinct subset of CD4⁺ T cells in the early 2000s marked a significant advancement in the understanding of the role of gut bacteria in mucosal immunity and inflammation [29, 30]. Subsequent studies revealed that the gut microbiota play a crucial role in shaping Th17 responses, with specific segmented filamentous bacteria shown to robustly induce Th17 differentiation in the intestinal lamina propria [31]. These insights established a strong link between microbial composition and immune regulation. Recently, this relationship has been extended to blood pressure regulation.

A report by Mell et al. [25] investigated the role of gut microbiota in blood pressure regulation using Dahl salt-sensitive (S) and salt-resistant (R) rats. Significant differences in microbial composition were found between the strains. Sequencing of 16S rRNA from cecal samples revealed that S rats had higher levels of bacteria of the family S24-7 of the phylum *Bacteroidetes* and the family *Veillonellaceae* of the phylum *Firmicutes* compared to R rats. Briefly, rats of both strains were kept on a high salt diet, and the microbiota were depleted with antibiotics. Next, each strain received transplanted microbiota from either S or R rats. The blood pressure in R rats was unaffected by the source of microbiota, however, S rats receiving R rat microbiota exhibited a persistent increase in systolic blood pressure and a reduced lifespan. This hypertensive effect was associated with a decrease in *Veillonellaceae* and an elevation in plasma acetate and heptanoate. Of note, the *Firmicutes/Bacteroidetes* ratio has been used as a readout of dysbiosis. Alterations have been associated with obesity, metabolic diseases, and hypertension [32, 33].

Notably, acetate modulates a hypotensive response, as acetate infusion leads to vasorelaxation and lowering of blood pressure [34, 35]. Therefore, elevated plasma acetate levels should be associated with lower blood pressure and not hypertension as reported by Mell et al. The authors suggested that variations in the expression levels of SCFA receptors or genetic differences between S and R rats might influence their responses to elevated circulating acetate. Further studies are necessary to elucidate the roles of SCFAs on blood pressure regulation in S and R rats. In humans, population studies could inform whether allelic variants within SCFA receptors correlate with differences in blood pressure regulation in hypertensive versus normotensive patients.

Bier et al. [36], studying S rats, showed that high salt intake increased fecal acetate, propionate, and isobutyric acid, while butyrate levels remained unchanged. However, the study did not inform whether these changes were mirrored in the plasma. Chakraborty et al. [8] employed targeted metabolomics to compare SCFA profiles in high-salt versus low-salt-fed rats and found no significant differences in circulating SCFA levels between high- and low-salt diet groups (despite microbiota shifts), emphasizing that fecal concentrations do not necessarily reflect systemic SCFA availability. Consistently, Yang et al. [37] showed that expression of the SCFA transporter Slc5a8 was downregulated in hypertensive S rats. While cecal butyrate levels were elevated, plasma concentrations were reduced, suggesting that impaired SCFA transport, rather than production, may limit their systemic bioavailability and blood pressure regulation responses.

It is well accepted that diets high in dietary fiber are associated with reduced risk for chronic disease, at least in part, because the SCFAs produced during the colonic fermentation of fiber beneficially influence the regulation of host physiology. Thus, could dietary fiber supplementation reduce blood pressure? The answer is complex since dietary fibers can impact host physiology in several ways. A recent meta-analysis by Faghihimani et al. [38], that included five randomized controlled trials and 233 participants, found that supplementation with dietary fibers such as inulin-type carbohydrates did not significantly reduce either systolic or diastolic blood pressure. However, when the groups were separated by sex, the analysis showed a reduction in systolic blood pressure among female participants. The authors concluded that more studies with hypertensive patients are needed to better evaluate the potential beneficial effects of prebiotics on blood pressure. In a different study, Becerril-Alacrón et al. [39] observed that supplementation with inulin led to a reduction in systolic blood pressure and prevented the increase in diastolic blood pressure in women with breast cancer undergoing chemotherapy. Thus, dietary fiber supplementation may help to promote a reduction in systolic blood pressure, especially in women, but the evidence is still limited, and the effects are not consistent across all populations. Further studies are necessary to evaluate the effects of dietary fiber supplementation on blood pressure regulation.

Interestingly, biological variables, such as sex and the circadian cycle, also may influence changes in microbiota and blood pressure regulation. Historically studied in isolation, the interplay between circadian rhythms and gut microbiota is now recognized as a critical component of blood pressure regulation. A report by Chakraborty et al. [40] demonstrated that changes in gut microbiota composition are linked to salt-sensitive hypertension and kidney damage. Using S rats subjected to low- and high-salt diets, they demonstrated that microbial communities fluctuated between active (dark) and rest (light) phases, mirroring changes in blood pressure patterns. Notably, specific bacterial taxa were associated with blood pressure rhythms, salt intake, and blood pressure dipping. Metagenomic analyses further revealed that microbial biosynthetic activity increased during the active phase, while catabolic pathways were more prominent during rest. These findings indicated that diurnal fluctuations in gut microbial metabolism may modulate the production or clearance of metabolites involved in blood pressure control.

Interestingly, a well-established relationship exists between night shift work and increased blood pressure and cardiometabolic disease, likely due to circadian disruption and its effects on the body's natural rhythms [41, 42]. A recent review by Lopez-Santamarina [32] explored how non-traditional work schedules impact the human gut microbiota and examined the potential role of probiotics in correcting dysbiosis. The authors suggested that probiotics may help maintain the diversity and stability of the gut microbiota in the unique environmental conditions associated with certain occupations. Although more targeted research is needed, current findings support the hypothesis that SCFA supplementation could be a promising strategy to mitigate blood pressure dysregulation associated with circadian disruptions in night shift workers.

Sex differences are increasingly acknowledged as key modifiers of gut microbiota and host physiology. Recent evidence has begun to uncover sex-specific relationships between gut microbiota and hypertension [43]. Sakamuri et al. [44] described that sex hormones, especially female sex hormones, have an impact on the gut microbiota. However, the underlying mechanisms driving sex-specific gut microbiota and hypertension remain largely unexplored. Bardhan et al. [45] identified a sex-specific mechanism by which the microbiota modulate the metabolism of tryptophan (an aromatic amino acid) and blood pressure via the indole pathway. Their findings suggest that salt affects tryptophan metabolism differently in males and females, potentially due to differences in microbial composition.

The literature discussed above indicates that gut microbiota play a key role in regulating blood pressure. It is likely that, at least in part, gut microbiota influence blood pressure regulation through metabolites produced and released into the circulation. In this regard, SCFAs stand out as beneficial metabolites that play a role in blood pressure regulation.

## SCFAs and gut microbiota

**Fig. 2:**
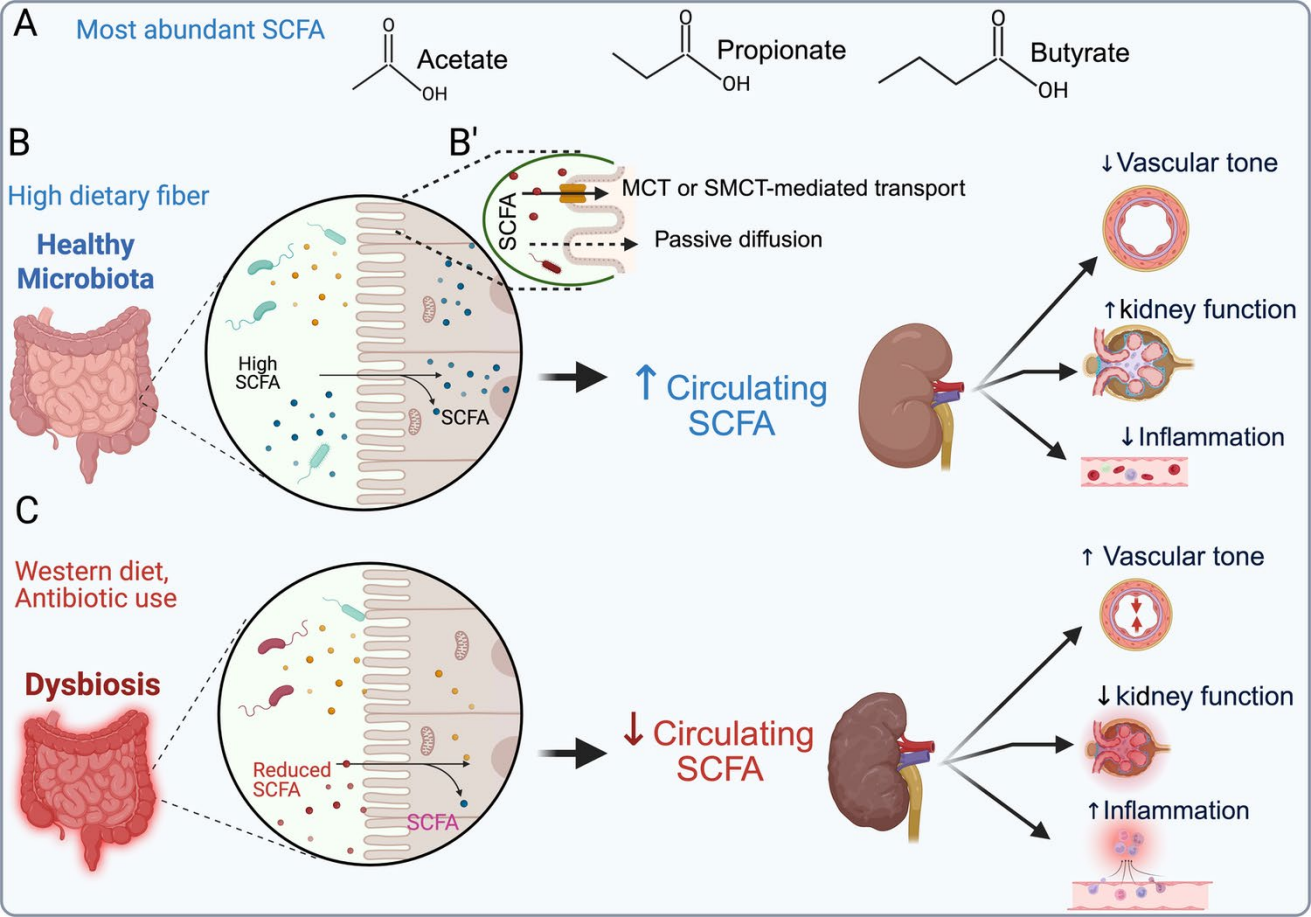
Circulating gut microbiota-derived SCFAs influence the immune system, kidney function, and vascular tone (**A**) The most abundant SCFAs (acetate, propionate, and butyrate) are produced by microbial fermentation of dietary fiber in the colon. (**B**) In the presence of a healthy microbiota supported by a high-fiber diet, SCFAs are produced in abundance. These SCFAs cross the intestinal epithelium via passive diffusion or active transport mediated by monocarboxylate transporters (MCTs) or sodium-coupled monocarboxylate transporters (SMCTs) (B′), leading to elevated circulating SCFA levels. Increased SCFAs positively impact kidney health by lowering vascular tone, reducing systemic and renal inflammation, and enhancing kidney function. (**C**) In contrast, a Western diet and excess antibiotic use may promote gut dysbiosis, resulting in reduced SCFA production and absorption. This leads to decreased circulating SCFAs, which in turn increase vascular tone, elevate inflammation, and impair kidney function

SCFAs are among the most abundant microbial metabolites in the gut that serve as critical mediators of host-microbe interactions [46, 47]. These molecules are primarily produced in the distal small intestine and colon by anaerobic bacteria fermenting dietary fibers. It is estimated that approximately 500–600 mmol of SCFAs are produced in the gastrointestinal tract daily. The production rates are contingent upon factors such as diet, microbial composition, and gut transit time. The concentration of SCFAs varies, ranging from 70 to 140 mM in the proximal colon and from 20 to 70 mM in the distal colon, as previously reviewed [48, 49]. Structurally, SCFAs are defined as simple carboxylic acids and classified according to their carbon chain length (i.e., SCFAs have fewer than six carbons). The most abundant SCFAs are acetate (two carbons), propionate (three carbons), and butyrate (four carbons) (Fig. 2A) [1, 47].

Once produced in the colon, SCFAs are absorbed by the intestinal epithelium via two primary mechanisms. First, passive diffusion allows SCFAs present in their uncharged, protonated forms within the colonic lumen to freely cross the apical membrane of colonocytes. Second, carrier-mediated transport facilitates SCFA uptake through specific transport proteins, including proton-dependent monocarboxylate transporters (MCT1/SLC16A1 and MCT4/SLC16A3) and sodium-coupled monocarboxylate transporters (SMCT1/SLC5A8 and SMCT2/SLC5A12) (Figs. 2B and 2 C) [50–52].

Following absorption, SCFAs enter the portal vein and travel to the liver, where a fraction of them is metabolized. The remaining SCFAs in the bloodstream are transported primarily in their free form, though some SCFA molecules may bind to albumin, facilitating delivery to peripheral tissues [49, 53].

While colonic SCFA concentrations can be found at > 100 mM [48, 54], circulating plasma levels are much lower and vary by species, diet, and metabolic health. In humans, fasting plasma concentrations typically range from 20–250 µM for acetate, 1–15 µM for propionate, and 0.3–2 µM for butyrate [55]. In mice, levels are generally as follows: acetate (50–200 µM), propionate (20–60 µM), and butyrate (5–15 µM), though values may vary with strain, diet, phase of light cycle, sex, feeding state, and analytical methods. Notably, these interspecies differences in SCFA levels and proportions underscore the need for caution when extrapolating findings from animal models to humans, particularly in studies of metabolism, immunity, and gut-organ communication.

Figure 2B illustrates the current model in which healthy microbiota, supported by a fiber-rich diet, lead to increased concentration of plasma SCFAs, reduced inflammation and vascular tone, and improved kidney function, resulting in better blood pressure control and a lower risk of CKD. In contrast, dysbiosis, exacerbated by factors such as a Western diet and antibiotics, results in reduced SCFA levels, elevated inflammation, increased vascular tone, kidney fibrosis, and a higher risk of CKD (Fig. 2C). While these physiological effects are clear, the molecular pathways underlying the impacts of SCFAs on kidney function remain poorly understood. Recently, increasing attention has been given to the roles of SCFAs as ligands for G protein-coupled receptors (GPCR) and their role in kidney function and blood pressure control [56–58]. The next sections will explore the effects of SCFAs and the activation of these receptors on kidney function and blood pressure regulation.

## SCFAs activate specific GPCRs to control blood pressure

In addition to serving as substrates for energy production and biosynthetic pathways, SCFAs act as receptor ligands. At the plasma membrane level, SCFA signaling events are mainly mediated through GPCRs. These receptors are found in several tissues, but the ones in the kidney and vasculature are thought to directly regulate blood pressure, while the receptors in immune cells indirectly influence blood pressure regulation via modulation of inflammatory responses.

Regarding blood pressure control, the most studied GPCRs responsive to SCFAs are GPR41, now known as free fatty acid receptor 3 (FFAR3, gene name *Ffar3*, also referred to as *Gpr41*), and GPR43 (FFAR2 protein, encoded by the gene *Ffar2*, also referred to as *Gpr43*). Additionally, GPR109A or HCA2 (Hydroxycarboxylic acid receptor 2), or NIACR1 (niacin receptor 1) also play a role in blood pressure regulation. GPR109A/HCA2 protein is encoded by the gene *Hcar2,* also referred to as *Gpr109a, or Niacr1*. Additionally, the olfactory receptors 78 (OLFR78) and OLFR558 have also been studied in the context of blood pressure regulation. The murine OLFR78 receptor is encoded by the gene *Or51e2* (olfactory receptor family 51 subfamily E member 2); *Olfr78* is often used as a synonym for this gene in mouse genetics studies. The OLFR558 is another olfactory receptor that may play a role in blood pressure control. OLFR558 is encoded by the *Or51e1* gene (olfactory receptor family 51 subfamily E member 1) also referred to as *Olfr558*. These gene names and synonyms are listed in the Mouse Genome Informatics database (MGI) and are interchangeably used in the literature.

**Fig. 3:**
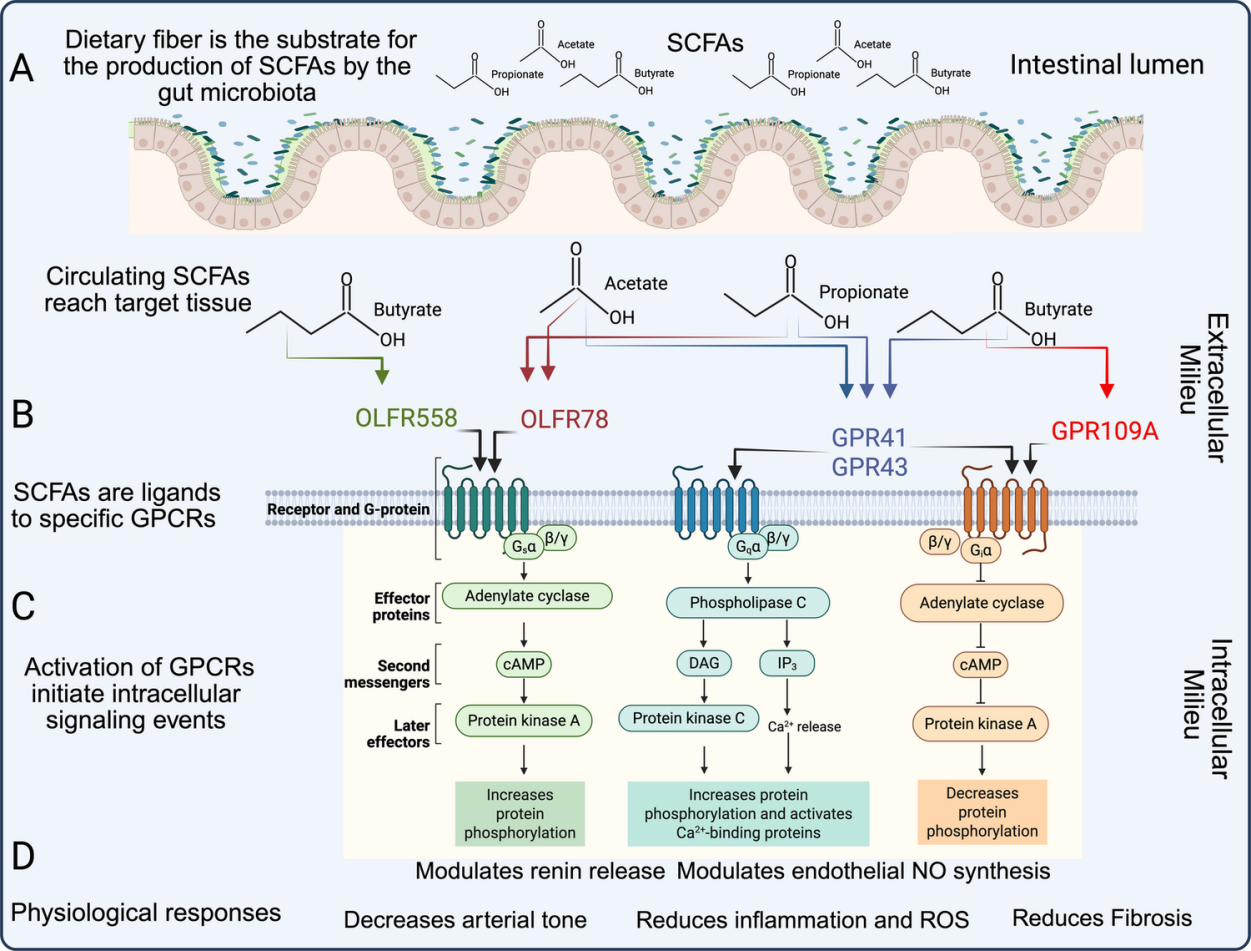
The action of SCFAs on specific cells is facilitated by the activation of GPCRs (**A**) Microbiota located in the lumen of the distal intestine and colon produce SCFA from ingested dietary fibers. (**B**) SCFAs are transported to target tissues where they act as ligands for specific GPCRs. (**C**) GPCR activation triggers intracellular signaling cascades through G-proteins (Gi, Gq, Gs) that modulate second messengers, including cAMP, IP3, and DAG. (**D**) These pathways regulate protein phosphorylation, calcium release, and several downstream effectors, resulting in physiological responses such as reduction of arterial tone, modulation of renin release, decreased inflammation and oxidative stress, and prevention of fibrosis

These receptors modulate inflammatory responses, epithelial barrier integrity, vasodilation, cell behavior, and energy metabolism, making them crucial for kidney function (Fig. 3) [57, 59–61]. Mechanistically, SCFAs produced by the microbiota (Fig. 3A) cross colonic cells to reach the circulation. Then they are transported to target tissues where they bind to GPCRs on cell membranes (Fig. 3B), triggering intracellular signaling cascades (Fig. 3C) that lead to physiological outcomes such as blood pressure regulation and nephron function (Fig. 3D).

The activation of these SCFA-GPCRs appears to be dependent on the SCFA structure, availability, and the receptor's binding affinity. Notably, EC50 values can vary depending on the species or the specific tissue/cell being studied. Circulating levels of acetate, propionate, and butyrate typically fall within the micromolar range, well within the activation thresholds of these receptors [62, 63].

The reported EC50 values for GPR41 range from 393 to 1072 µM for acetate, 6 to 127 µM for propionate, and 33 to 158 µM for butyrate. In contrast, GPR43 exhibits EC50 values ranging from 35 to 431 µM for acetate, 14 to 290 µM for propionate, and 28 to 371 µM for butyrate. These receptors can also respond to other SCFAs, though typically with lower affinity [64, 65]. GPR109A is activated by both niacin and butyrate. Niacin can bind and activate GPR109A [66]. However, butyrate can also bind and activate GPR109A and is accepted as the physiological ligand [61]. OLFR78 is mainly activated by acetate (EC50 of 2.01 mM to 2.35 mM), and propionate (EC50 of 0.63 to 0.92 mM) [67, 68]. Conversely, OLFR558 is activated by butyrate with an EC50 of ~ 0.2 mM. These measurements indicate that physiological changes in SCFA concentrations could potentially influence SCFA-mediated G protein-coupled receptor signaling and host physiology.

In terms of intracellular signaling pathway activation, the same ligand-receptor pair may activate different intracellular pathways in different cells (Table 1; Figs. 3A and 3B). When stimulated by SCFAs, GPR43 activates downstream signaling through both Gi/o (inhibitory G-protein/olfactory G-protein subunits) and Gq (which activates phospholipase C). Activation of the Gi/o pathway by GPR43 suppresses cyclic adenosine monophosphate (cAMP) production, leading to activation of extracellular signal-regulated kinases (ERK) and reduced protein kinase A (PKA) activity. In contrast, Gq signaling elevates intracellular calcium (Ca^2^⁺) levels, activates phospholipase C (PLC), and subsequently activates mitogen-activated protein kinase (MAPK) pathways (Figs. 3B and 3 C) [49, 63]. Internalization of GPR43 signaling is modulated by β-arrestin, a key negative regulator of GPCR activity. For instance, β-arrestin-2 binding to GPR43 diminishes its anti-inflammatory effects by inhibiting NF-κB signaling [69].

**Table 1:** Key characteristics of main SCFA-binding GPCRs: ligands, pathways, and tissue distribution

Receptor	Gene Name	SCFA Ligand	Signaling Pathway	Tissues of Expression	References
Gpr41	*Ffar3* (m) *Gpr41*(m) *FFAR3* (h)	Acetate Propionate Butyrate Formate	Gi/o, α-gustducin	Kidney Intestine Adipose tissue Spleen Immune cells Pancreas Stomach Lung Heart Brain	Brown et al. [65] Le Poul et al. [64] Eberle et al. [71]
Gpr43 Gpr43/Gpr41 heterodimer	*Ffar2* (m) *Gpr43* (m) *FFAR2* (h)	Acetate Propionate Butyrate Formate Pentanoate	Gi/o, Gq, β-Arrestin-2	Kidney Intestine Adipose Skeletal muscle Immune cells Spleen Pancreas	Brown et al. [65] Le Poul et al. [64] Zhao et al. [145] Smith et al. [146] Lee et al. [69] Macia et al. [147] Ang et al. [73]
Gpr109a	*Hcar2* (m) *Niacr1*(m) *Gpr109a* (m) *HCAR2* (h)	Butyrate Niacin	Gi/o, β-Arrestin-1 Arrestin-2 Arrestin-3	Kidney Immune cells Intestinal lumen Adipose Liver	Thangaraju et al. [74] Felizardo et al. [84] Walters et al. [148] Yang et al. [75] Li et al. [77] Yadav et al. [149]
Olfr78	*Psgr* (m), *Olfr78* (m) *Or51e2* (m) *OR51E2* (h)	Acetate Propionate	Gαs Unknown	Kidney Prostate Colon Lung	Pluznick et al. [67] Aisenberg et al. [150] Fleischer et al. [151] Poll et al. [93]
Olfr558	*Olfr558* (m) *Or51e1* (m) *OR51E1* (h)	Butyrate	Gαs	Kidney Aorta Heart	Xu et al. [57] Brunskill et al. [81]

Table 1 provides an overview of key G protein-coupled receptors (GPCRs) that respond to short-chain fatty acids (SCFAs), detailing their alternative names, ligand specificity, signaling pathways, tissue distribution, and supporting references. The SCFAs considered include acetate, propionate, butyrate, formate, and pentanoate. The receptors listed are GPR41 (also known as FFAR3, Free Fatty Acid Receptor 3), GPR43 (FFAR2, Free Fatty Acid Receptor 2), GPR109A (also referred to as HCAR2, Hydroxycarboxylic Acid Receptor 2, or NIACR1, Nicotinic Acid Receptor 1), Olfr78 (Olfactory receptor 78, known as PSGR, Prostate-Specific G protein-coupled receptor, in mice and olfactory receptor family 51 subfamily E member 2 (OR51E2) in humans) and Olfr558 (Olfactory receptor 558, also known as olfactory receptor family 51 subfamily E member 1 (OR51E1) in humans). These receptors activate a variety of intracellular signaling cascades, including Gi/o (inhibitory G-protein subunits), Gq (which activates phospholipase C), and Gαs (which activates adenylyl cyclase), as well as β-arrestin-1, β-arrestin-2, arrestin-2, arrestin-3, and α-gustducin, a G-protein subunit involved in taste receptor signaling. These GPCRs are expressed across multiple tissues and organs, including the kidney, intestine, adipose tissue, spleen, immune cells, pancreas, stomach, lung, heart, brain, skeletal muscle, colon, liver, and prostate. References listed alongside each receptor provide supporting evidence for their ligand specificity, signaling mechanisms, and tissue-specific expression

Activation of GPR41 suppresses cAMP production via Gi/o and promotes phosphorylation of ERK1/2 (Figs. 3B and 3 C). Interestingly, GPR41 responds primarily to longer SCFAs such as propionate, butyrate, and valerate. Kimura et al. [70] demonstrated that SCFAs and ketone bodies directly regulate the sympathetic nervous system through activation of GPR41. This receptor appears to be highly expressed in sympathetic ganglia in both mice and humans. While propionate activated sympathetic outflow through GPR41, the ketone body β-hydroxybutyrate, elevated during fasting or diabetes, inhibited sympathetic nervous system activity by antagonizing GPR41. Mechanistically, GPR41-mediated activation of sympathetic neurons involved Gβγ-PLCβ-MAPK signaling pathways. Importantly, these opposing effects of SCFAs and ketone bodies on sympathetic nervous system activity were closely linked to their influence on energy expenditure, highlighting a critical role for GPR41 in balancing metabolic homeostasis.

Eberle et al. [71], using molecular and immunohistochemical assays, showed that a special area of the rodent stomach (the fundus) contains brush cells that express GPR41 and GPR43, which are activated by SCFA. Interestingly, these GPR41 receptor proteins activate intracellular signaling through α-gustducin at the apical brush border of these cells. Hoon et al. [72], reported that α-gustducin has a high degree of homology to the Gi/o family and can bind GPCRs.

GPR41 can also form a heterodimer with GPR43, resulting in unique signaling characteristics. This GPR41/43 heterodimer enhances intracellular calcium signaling by approximately 1.5 times relative to GPR43 alone and significantly increases β-arrestin2 recruitment, roughly about 30 times higher than GPR41 alone. Interestingly, the heterodimer loses the capacity to inhibit cAMP production [73].

GPR109A is a Gi/o-coupled GPCR found in adipose tissue, immune cells, and the epithelial cells of the colon and kidney. Although originally identified as the receptor for niacin, butyrate and β-hydroxybutyrate are also physiological ligands, while acetate and propionate do not appear to activate this receptor [66, 74–76]. Internalization and desensitization of this receptor also involve the recruitment of arrestins (arrestin-3) [77]. Also, β-Arrestin1/2 have been shown to act as regulators of GPR109A activation and degradation in myeloid cells [75].

Both *Olfr78* and *Olfr558* encode murine olfactory chemosensory receptors broadly expressed across various tissues beyond the olfactory epithelium. Their human counterparts are *OR51E2* and *OR51E1*, respectively. In the olfactory system, these receptors couple with Go proteins, while in non-olfactory tissues, they signal through Gαs proteins (which activate adenylyl cyclase), leading to increased intracellular cAMP levels (Figs. 3B and 3 C) [56]. OLFR78 and its human ortholog (OR51E2) respond to acetate and propionate, but not butyrate, with EC50 of 2.35 mM for acetate and 920 μM for propionate. OLFR78 is found in kidney blood vessels, vascular smooth muscle cells, and the intestinal tract [67]. Functionally, propionate stimulates renin release from isolated glomeruli in wild-type mice, an effect absent in *Olfr78* knockout mice [67]. Additionally, OLFR78 signaling mediates an acute blood pressure-lowering response to propionate in wild-type mice, a response that is lost in the knockout mice [78].

OLFR558 was identified as an SCFA receptor for butyrate, with an EC50 value of 0.51 mM in murine kidney cortex [79]. OR51E1, the human ortholog, stimulates adenylyl cyclase in response to treatment with SCFAs (except for acetate) or medium-chain fatty acids [80]. *Olfr558* mRNA was found enriched in renin cells [81]. However, its importance in these cells remains elusive; recent evidence suggests that this receptor may be, in part, responsible for sex-related differences in blood pressure [82].

Activation of these SCFA-GPCR signaling pathways is responsible for changes in cellular behavior, which impact kidney function and blood pressure control (Fig. 3D).

## Tissue-specific expression of SCFA-GPCR related to blood pressure regulation

Multiple tissues exhibit expression of GPCRs that respond to SCFA (Table 1). We will further examine the evidence of SCFA activating GPCR signaling in the kidneys, blood vessels, and immune cells, focusing on their role in regulating blood pressure. *Gpr41*, *Gpr43*, and *Olfr78* expressions are found in the main blood vessels (renal artery, aorta, and iliac artery). Specifically, *Gpr41* and *Olfr78* are enriched in the renal artery. *Olf78* receptor is expressed in smooth muscle cells of the vasculature, including the major branches of the renal artery and the juxtaglomerular afferent arteriole.

Additionally, *Olfr78* is expressed in smooth muscle cells of small blood vessels of several tissues, including the heart, diaphragm, skeletal muscle, and skin. In the heart, esophagus, and stomach, *Olfr78* is also expressed in axons of autonomic neurons and neurons of the enteric plexus [67, 78]. *Olfr558* is expressed in renin-positive cells in the kidney and vascular smooth muscle cells, where it has been suggested to play an evolutionarily conserved role in mediating sex differences in blood pressure [81, 82]. Immunohistochemical analyses of human kidney biopsy specimens revealed the presence of GPR41 and GPR43 protein in the distal renal tubules and collecting tubules [60]. Finally, GPR109A has been found in adipose tissue near the kidney in low amounts, and its role in kidney disease is still unknown [83]. GPR109A has been identified in podocytes, where it seems to regulate inflammation [84]. Additionally, B-cell-specific GPR109A has been shown to limit humoral immune response in kidney disease [85].

## SCFA signaling in kidney and systemic physiology

The “gut-kidney axis” is a bidirectional communication network that underscores microbiota's role in kidney physiology and systemic health [86, 87].

Healthy gut microbiota have been associated with kidney health, while reduced levels of SCFA-producing bacteria have been observed in individuals with CKD [3, 24, 86]. As reviewed by Li et al. [88], SCFAs regulate multiple pathways critical for kidney development and function. These include immune modulation, antioxidant defense, suppression of fibrosis, and blood pressure control. Most of these effects appear to be mediated through GPCR signaling, and thus, understanding the roles of SCFA-GPCR in kidney physiology and blood pressure regulation holds therapeutic potential.

Initial evidence for SCFA-GPCR signaling in the kidney came from Brown et al. [65] and Le Poul et al. [64], who confirmed the expression of GPR41 and GPR43 in the kidney and several other tissues and identified SCFAs as their ligands. Similarly, Nakai et al. [89] reported a decreased mRNA expression of GPR41*,* GPR43*,* and GPR109A in hypertensive human subjects, but only GPR43 levels remained significantly lower after adjusting for age, sex, and BMI. Consistent with this, removal of *Gpr41* in mice led to elevated systolic blood pressure [58].

Interestingly, while acetate and butyrate levels are associated with hypotensive effects, the cohort of hypertensive patients in Nakai’s study had higher plasma levels of these SCFAs than normotensive controls. This paradoxical finding may be explained by the observed reduced expression of corresponding SCFA-GPCRs in the hypertensive subjects and perhaps reduced signaling. Possibly, tissue-specific differences in receptor expression or gene polymorphism may contribute to hypertension in humans. Notably, in Nakai’s study, they measured mRNA in white blood cells only, leaving the status of important sites for blood pressure regulation (endothelial cells and kidneys) unexamined.

In a recent study, Felizardo et al. [84] demonstrated that GPR109A, a receptor activated by butyrate, is expressed in podocytes. To explore its role in kidney injury, the authors induced nephropathy in *Gpr109a* knockout and wild-type mice using a single dose of adriamycin, followed by treatment with either sodium butyrate or a diet enriched in butyrate-releasing high-amylose maize starch. In wild-type mice, butyrate treatment significantly reduced proteinuria, preserved podocyte integrity at the glomerular basement membrane, and attenuated both glomerulosclerosis and kidney inflammation. These protective effects were absent in *Gpr109a*-deficient mice, indicating that GPR109A function is essential for butyrate-mediated kidney protection. These findings underscore the importance of studying tissue- and cell-specific receptor expression and function, particularly in the context of hypertension and kidney disease.

In this context, Muralitharan et al. [90] generated a double knockout mouse model lacking both *Gpr41* and *Gpr43*, and observed that loss of SCFA receptor signaling increased susceptibility to hypertension and promoted cardiorenal fibrosis and hypertrophy. The absence of *Gpr41/43* impaired gut barrier integrity and facilitated the translocation of bacterial endotoxins like lipopolysaccharide (LPS) into circulation, which triggered kidney macrophage infiltration and pro-inflammatory cytokine production. Blocking LPS signaling through TLR4 inhibition successfully rescued the cardiovascular abnormalities in knockout mice. While *Gpr41/43* were partially responsible for the blood pressure-lowering and cardio-protective effects of a high-fiber diet, improvements in gut barrier function and kidney macrophage infiltration occurred independently of these receptors. Interestingly, analysis of UK Biobank data revealed that genetic variants linked to reduced expression of *Gpr41/43* are more common in hypertensive individuals, supporting their potential as therapeutic targets in blood pressure control.

In addition to GPR41 and GPR43, OLFR78 is activated by SCFAs and is also found in the kidneys. Pluznick et al. [67] reported that *Olfr78* RNA is expressed in the juxtaglomerular apparatus and vascular smooth muscle of small resistance arteries. Activation of this receptor by propionate stimulated renin secretion, promoting vasoconstriction and elevating blood pressure.

However, propionate infusion induces relaxation and hypotensive effects in wild-type mice. To resolve this apparent conundrum, Pluznick et al. [67] took advantage of antibiotic treatment to reduce microbiota output and knockout models to dissect the effects of SCFA and OLFR78 activation on blood pressure regulation. Their findings revealed an elegant balancing mechanism: while activation of OLFR78 promotes renin release, GPR41 activation counteracts it by inducing vasodilation, suggesting these receptors may have a hierarchical response to maintain blood pressure homeostasis. Pluznick [78] proposed that while propionate activates both GPR41 and OLFR78, which have opposing effects on blood pressure, the presence of dual signaling may serve a regulatory function. GPR41 has a lower EC50 (~ 274 µM), and is more sensitive and likely active at normal plasma propionate levels, promoting vasodilation and lowering blood pressure. In contrast, OLFR78 has a higher EC50 (920 µM), becoming active only at elevated propionate concentrations. Thus, OLFR78 acts as a counterbalance or “brake” to prevent excessive drops in blood pressure when propionate levels rise. Supporting this idea, mice lacking *Olfr78* exhibit an exaggerated hypotensive response to propionate, confirming the receptor’s role in maintaining blood pressure stability.

Further supporting this dual role, Weber et al. [91] found that angiotensin II treatment upregulated *Olfr78* and downregulated *Gpr41 and Gpr43* in mouse kidneys. Mechanistically, the expression of specific microRNAs (*miR-132* and *miR-329*) that target *Gpr41 and Gpr43* is increased upon angiotensin II treatment, leading to a reduction of expression of the targeted GPCRs and favoring an increase in blood pressure [92].

A recent study by Poll et al. [93] confirmed that *Olfr78* is expressed in the juxtaglomerular apparatus and peripheral vasculature and is responsive to SCFA treatment. Using radiotelemetry, the authors found that *Olfr78*-deficient mice did not differ in baseline blood pressure or in responses to salt intake, although they exhibited lower renin levels and reduced heart rates on high-salt diets. These results suggest that OLFR78 activation may not regulate basal blood pressure but may be critical for fine-tuning renin responses during hypotensive challenges or elevated SCFA exposure.

GPR109A is another GPCR activated primarily by butyrate. Li et al. [94] used dietary fiber and SCFA supplementation in streptozotocin-induced diabetic mice lacking *Gpr4*3 or *Gpr109a* to investigate the role of these receptors in diabetes-induced kidney injury. High-fiber diets and SCFA treatments reduced albuminuria, glomerular hypertrophy, and interstitial fibrosis, but these protective effects were absent in receptor-deficient mice. SCFA treatment also reduced inflammatory and fibrotic gene expression in tubular cells and podocytes under hyperglycemic conditions. These findings underscore that GPR43 and GPR109A are essential mediators of SCFA-driven renoprotection, particularly in diabetic nephropathy.

In line with these thoughts, in a later study using a type 2 diabetes model (db/db mice), Snelson et al. [95] found that a diet enriched in resistant starch (12.5%) significantly reduced albuminuria, improved SCFA profiles (acetate, propionate, and butyrate), and lowered gut permeability and kidney inflammation, without affecting kidney fibrosis. These effects were attributed to enhanced SCFA production and suggested a therapeutic role for dietary fiber in metabolic kidney disease.

Using multiple imaging techniques, Ang et al. [73] investigated a physical interaction between GPR41 and GPR43. The authors found that these receptors can form heterodimers, at least in human monocytes, macrophages, and HEK293 cells. The GPR41 and GPR43 heteromer exhibits enhanced signaling, including a 1.5-fold increase in cytosolic Ca^2^⁺ and a 30-fold increase in β-arrestin-2 recruitment compared to the individual receptors. This enhanced signaling is sensitive to Gpr43 antagonism and G protein inhibition. Unlike the homomeric receptors, the heteromer does not inhibit cAMP production but instead uniquely activates p38 MAPK phosphorylation. These findings suggest the heterodimer has signaling properties distinct from its components. This receptor interaction presented a novel mechanism by which GPCR-mediated SCFA signaling can impact physiology. Targeting the GPR41-GPR43 heterodimer formation may provide new opportunities for drug development targeting metabolic and inflammatory diseases.

Lastly, *Olfr558*, which is closely related to *Olf78*, also appears to be involved in blood pressure regulation. Brunskill et al. [81] employed microarray screenings to detect genes defining renin cell identity and showed that *Olfr558* is enriched in renin cells, and its expression increased in mice treated with captopril (an antihypertensive drug that inhibits the angiotensin-converting enzyme).

Xu et al. [57] investigated the roles of the receptor OLFR558 and its human ortholog OR51E1, in the vasculature. Using real-time cAMP and calcium imaging, the authors found that butyrate activated both OLFR558 and OR51E1 in a dose- and time-dependent manner, increasing cAMP and Ca^2^⁺ responses in HEK293T cells. Isobutyrate did not raise cAMP but did trigger Ca^2^⁺ responses, suggesting that distinct signaling pathways may be activated by different ligands. *Olfr558* expression was found specifically in vascular smooth muscle of the kidney, heart, and aorta. After angiotensin II infusion, *Olfr558* expression increased in the heart, but not in the kidney. These results suggest OLFR558 is a receptor that links gut microbiota activity to cardiovascular function.

Understanding these receptor-specific pathways is crucial for developing targeted therapies that modulate SCFA signaling to attenuate kidney inflammation, preserve epithelial integrity, and control blood pressure. However, supraphysiologic SCFA doses can trigger immune dysregulation and kidney inflammation, highlighting the need for precision in SCFA-based interventions, as shown by recent work [96].

## SCFAs and vascular tone regulation

The regulation of vascular tone through vasoconstriction and vasodilation is a critical determinant of blood pressure. Remarkably, the first evidence of an SCFA influencing vascular relaxation dates to 1928, when Bauer and Richards identified acetate as a vasodilator [35]. Subsequent research has confirmed that infusion with SCFAs elicited a dose-dependent relaxation response [65, 97]. Additionally, studies in the 1980 s showed that acetate, commonly used as a buffer source in hemodialysis, contributes to relaxation and hypoxemia observed during the procedure [98–100].

Notably, other SCFAs also present cardiovascular effects. In models of angiotensin II-induced hypertension, Marques et al. [34] demonstrated that propionate supplementation attenuated vascular dysfunction and cardiac hypertrophy, underscoring its protective cardiovascular effects. Similarly, Poll et al. [97] reported that acetate administration reduced blood pressure and heart rate in rats.

A study by Knock et al. [101] found that propionate induced relaxation in rat mesenteric arteries through the action of endothelium-derived hyperpolarizing factor (EDHF). Interestingly, this vasorelaxation was blocked by thapsigargin, an inhibitor of the sarcoplasmic/endoplasmic reticulum calcium ATPase (SERCA) pump. These findings suggested that propionate treatment can mobilize intracellular calcium stores and facilitate EDHF-mediated vasodilation.

Joe et al. [102] showed that germ-free rats exhibit reduced blood pressure and impaired vascular contractility compared to those colonized with microbiota from pathogen-free rats. The authors showed that colonization restored blood pressure and vascular tone, implicating a microbiota-dependent, vascular-mediated mechanism of blood pressure regulation. Mechanistically, this effect was linked to changes in actin cytoskeletal remodeling, evidenced by increased cofilin phosphorylation upon colonization.

Interestingly, Karbach et al. [103] found that germ-free mice were protected against angiotensin II–induced vascular and kidney inflammation. However, upon microbial colonization, these mice developed endothelial dysfunction, increased MCP-1 and IL-17 signaling, and higher immune cell infiltration, all contributing to vascular inflammation, hypertension, and organ damage.

As discussed above, colonization of the mouse small intestine with specific bacterial strains such as segmented filamentous bacteria (SFB) alone is enough to trigger the development of Th17 cells, CD4⁺ T cells that secrete IL-17 and IL-22, in the lamina propria. SFB colonization is known to upregulate genes linked to inflammation and antimicrobial defense [31]. Therefore, targeting microbiota-regulated immune pathways could offer new strategies for boosting mucosal immunity and managing hypertension in individuals with dysbiosis.

Importantly, the vascular effects of the microbiota may also be sex-specific. Edwards et al. [104] assessed vascular function in male and female germ-free mice and found impaired arterial contraction in both sexes, but with more severe vascular remodeling in males. Germ-free males showed increased arterial stiffness and inward hypotrophic remodeling, while females exhibited outward hypertrophic changes, resembling age-related vascular alterations. Moreover, neutrophil-derived reactive oxygen species (ROS) were elevated in males but suppressed in females, indicating that the microbiota modulates vascular health in a sex-dependent manner.

Collectively, these studies highlight SCFAs as key regulators of vasodilation and emphasize the gut microbiota’s influence on vascular structure and function. SCFAs contribute to endothelial relaxation, immune modulation, and cytoskeletal remodeling, making them essential to blood pressure homeostasis.

## SCFAs and kidney development

Most circulating SCFAs are produced through the fermentation of dietary fiber by the maternal gut microbiota. The SCFAs that reach the fetus are derived primarily from the mother’s microbiome. It is unlikely that the fetal microbiota contribute to fetal SCFA levels, as the fetal gut is not sufficiently developed during the critical window of kidney development. There remains considerable debate over whether microbial colonization of the fetal intestinal tract occurs in utero, and if so, at what developmental stage this takes place [105]. Analysis of meconium samples collected from the small intestine of mid-gestation fetus showed extremely low bacterial load near the limits of detection, nonetheless, some of the identified bacteria appeared to have immuno-modulatory capacity [106]. Although there is no strong evidence linking fetal intestinal microbes directly to fetal kidney development, it is plausible that maternal microbiota-derived metabolites can cross the placenta and impact nephrogenesis.

While SCFAs are well-studied for their roles in blood pressure regulation, emerging evidence suggests SCFA balance may play an important role in kidney development and may help mitigate developmental complications, such as oligonephropathy and hypertension. Beyond their hemodynamic effects, SCFAs appear to influence fetal nephrogenesis.

Gestational dysbiosis, often observed in conditions like preeclampsia, has been linked to adverse pregnancy outcomes, including premature birth and reduced nephron endowment [107–111]. Although the direct effects of maternal dysbiosis on fetal kidneys remain under-explored, it is plausible that altered SCFA production during gestation may influence kidney development.

Kidney development is a tightly regulated process involving the expansion and differentiation of nephron progenitor cells, as well as the establishment of epithelial integrity [112]. Since the final nephron number at birth correlates with long-term blood pressure control, disruptions to this developmental trajectory can have lasting health consequences [113, 114]. Recent research shows that SCFAs, particularly acetate, may influence nephron endowment.

A key study by Diniz et al. [12] demonstrated that acetate supplementation in embryonic kidney explants increased nephron progenitor cell mass without affecting nascent nephron formation. This suggests that SCFAs can modulate progenitor cell dynamics and highlights the importance of circulating SCFA levels for proper nephrogenesis. Notably, analysis of proteomic and transcriptomic data from E13.5 nephron progenitor cells revealed minimal or undetectable expression of SCFA-activated GPCRs, including GPR41, GPR43, GPR109A, OLFR78, and OLFR558 [12]. Thus, the impact of acetate on these early nephron progenitor cells does not appear to be modulated by SCFA-GPCR signaling but rather by intracellular acetate metabolism.

Interestingly, metabolic acidosis is a common condition in both preterm and term newborns and often necessitates base therapy, such as acetate or bicarbonate supplementation. In premature infants with pH imbalances, clinicians may administer sodium acetate as part of parenteral nutrition to support acid–base balance and mitigate the risk of acidosis [115, 116]. Acetate supplementation provides dual advantages, correcting metabolic acidosis while also potentially enhancing nephrogenesis in extremely premature infants. The latter is an active area of research in the Tortelote Laboratory at Tulane University.

A recent study by Dang et al. [117] showed that the maternal microbiome profoundly influences offspring development through stem cell programming. In fetal stem cells, maternal microbiota with greater metabolic output, including higher SCFA production, activated critical signaling pathways, such as mTOR. This metabolic loop may shape proliferation and differentiation, influencing tissue development and long-term physiological outcomes.

Applying this to nephrogenesis, it is reasonable to propose that maternal microbiome diversity could shape nephron progenitor cells’ proliferation and differentiation, potentially altering nephron number. Disruptions to this system may contribute to oligonephropathy and the long-term risk of CKD or hypertension. As such, preserving maternal microbiome health through diet, prebiotics, or probiotic supplementation may represent a viable strategy to optimize fetal kidney development during gestation.

## Antibiotics in pregnancy and their impact on SCFA production and blood pressure regulation

Antibiotic therapy stands out as one of the most significant medical advancements of the past century, and it constitutes roughly 80% of all medications prescribed during pregnancy [118]. Notably, excessive and inappropriate use of antibiotics has led to serious challenges, including the emergence and spread of antibiotic-resistant bacteria, which pose a growing threat to global health [18].

Only a few studies have examined the direct impact of antibiotics on maternal microbial ecosystems or the long-term consequences of prenatal exposure. The use of antibiotics impacted the host’s gut microbiota and induced metabolic changes during pregnancy [119, 120]. Notably, the long-term impacts on the health of both mothers and offspring are still unknown. This stands as a major gap in our understanding of how antibiotics might alter fetal development, particularly during sensitive windows such as kidney organogenesis.

Antibiotic-induced dysbiosis disrupts epithelial barrier integrity and may alter maternal–fetal signaling, with potential long-term consequences for the offspring [17, 18, 121]. The maternal gut microbiota play a critical role in SCFA production, which may influence fetal development [120]. Antibiotic use during pregnancy can shift microbial composition and suppress SCFA-producing bacteria [17, 109], potentially impacting nephrogenesis. These alterations may result in reduced nephron progenitor cell populations, predisposing the offspring to hypertension and CKD later in life [121, 122].

Pluznick et al. [61, 67] showed that antibiotic treatment decreased SCFA-producing microbes, altering kidney physiology and blood pressure regulation, specifically in an *Olfr78* null background. In a recent study, Muralitharan et al. [90] demonstrated that gestational dysbiosis increased intestinal permeability, allowing LPS and other microbial toxins to leak into the bloodstream, triggering kidney inflammation and fibrosis.

Galla et al. [123] used a hypertensive rat model to test the hypothesis that antibiotic use in early life can reshape gut microbiota and impact blood pressure regulation throughout life. The authors conducted two experiments in which they altered the gut microbiota of young, genetically hypertensive rats and dams during gestation and lactation. Amoxicillin treatment reduced the *Firmicutes/Bacteroidetes* ratio and lowered blood pressure, both in young rats and in offspring of treated dams. Of note, the reduction in blood pressure correlated with decreased levels of *Veillonellaceae*, which is a succinate-producing bacterial family, and with reduced serum succinate levels, previously linked to hypertension [124].

Notably, an increased *Firmicutes/Bacteroidetes* ratio reflects an increased capacity to ferment dietary polysaccharides to SCFA [19, 33]. The phylum *Firmicutes* includes many known SCFA producers [19, 37, 125]. SCFAs contribute to numerous physiological processes and are increasingly recognized as important regulators of blood pressure, potentially offering key insights into the understanding and treatment of hypertension in both adults and children.

These findings underscore the importance of preserving maternal microbial integrity during pregnancy. Early interventions, such as probiotics, prebiotics, and SCFA supplementation, may help restore microbial balance and protect fetal kidney development.

## Impacts of gut microbiota-derived SCFAs on pediatric kidney disease

Pediatric hypertension and loss of kidney function are increasingly being acknowledged as global health concerns. While updated guidelines have been introduced to support clinical management, the underlying causes of their rising prevalence in children remain unclear [126]. Genetic predisposition and environmental influences alone do not sufficiently explain this trend.

Given the established connection between early-life gut microbial stability and long-term health outcomes, it is plausible that alterations in gut microbiota during early development may influence blood pressure regulation in children. Moreover, emerging clinical and experimental studies point to gut microbiota dysbiosis as a potential contributor to early-onset kidney disease [27, 127].

**Fig. 4:**
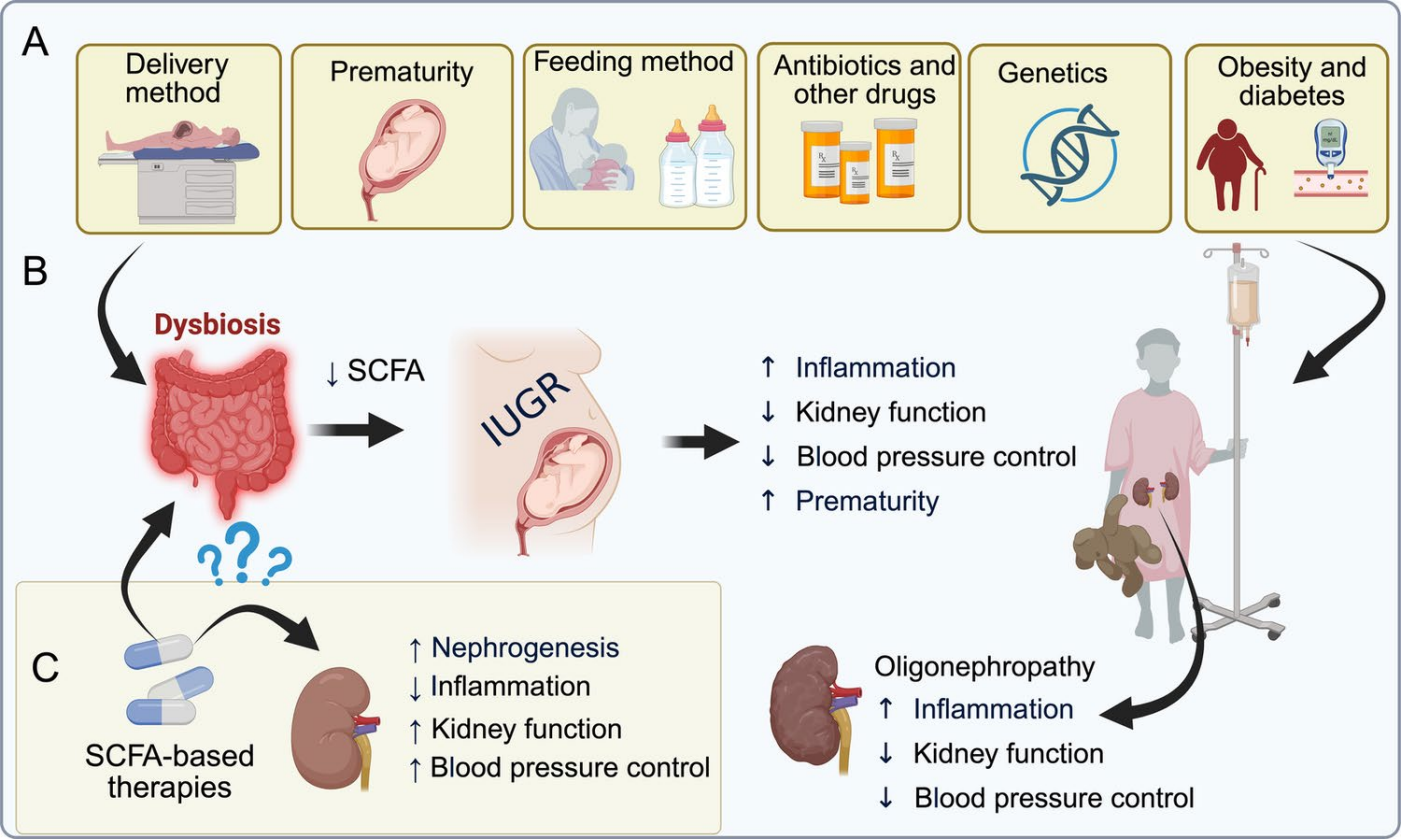
Rationale for therapeutic use of SCFA to prevent dysbiosis and pediatric CKD (**A**) Gut dysbiosis and pediatric kidney disease share several risk factors (e.g., prematurity, genetics, maternal diabetes, obesity). (**B**) Dysbiosis leads to a reduction in bacteria-mediated fermentation of dietary fiber and SCFA production, which is correlated with decreased maternal kidney function, increased blood pressure, and inflammation and intrauterine growth restriction. On the fetal side, these factors correlate with oligonephropathy, loss of kidney function, increased inflammation, and impaired blood pressure control. (**C**) SCFA-based therapy may support proper kidney development and function. Also, SCFA supplementation may help to reduce inflammation and to maintain good blood pressure control

Poor postnatal growth and intestinal dysbiosis are common in extremely preterm infants and are each linked to long-term health complications. Several factors that affect infants’ microbiota composition, such as prematurity, obesity, exposure to nephrotoxic medications, breast vs. formula feeding, genetic predisposition, and metabolic disorders, are also recognized risk factors for kidney disease (Figs. 4A and 4B) [17, 113, 119, 121, 128, 129].

Emerging evidence highlights how different feeding methods influence SCFA profiles in early life and suggests a potential link with healthy development. Acetate is the predominant SCFA in the feces of breastfed infants. In contrast, propionate levels are low, and butyrate is nearly undetectable in these breastfed infants, but can be detected in those infants fed formula, indicating the establishment of different intestinal microbiota and fermentation profile [130]. Since SCFAs may play important roles in kidney development and function, alterations in their levels, whether due to maternal health, prematurity, or feeding composition, may have lasting effects on a child’s kidney and cardiovascular health. These insights support the hypothesis that therapeutic modulation of SCFA levels may offer a promising strategy to prevent or mitigate kidney disease and hypertension (Figs. 4B and 4 C).

Furthermore, research involving premature infants kept in incubators indicates a critical period between the second and third weeks of life, during which fecal butyrate levels surge. Excessive production of butyrate, or the bacteria responsible for its production, has been linked to gastrointestinal problems like necrotizing enterocolitis, a significant risk for these infants [131]. Perhaps, more than gastrointestinal problems, the differences in SCFA profile in premature vs. at-term infants and breast milk-fed vs. formula-fed infants may also impact postnatal development and function of several organs, for instance, the developing kidneys.

Pediatric kidney diseases span a diverse spectrum of conditions that can significantly impact long-term health, increasing the risk of morbidity and mortality in adulthood [132]. Growing evidence supports the idea that many adult kidney disorders have developmental origins in fetal or early postnatal life [122, 133].

In a cohort of 105 pediatric CKD patients, Lu et al. [134] examined associations between plasma SCFA levels and cardiovascular health. Among 65 children over six years of age followed for one year, 27.7% developed hypertension. Notably, plasma acetate increased in children with stable blood pressure, while it remained unchanged in those whose blood pressure worsened. Higher baseline plasma butyrate was associated with a greater risk of blood pressure elevation, and both groups experienced a decline in butyrate over time. Elevated baseline propionate was also linked to reduced cardiac ejection fraction. These findings suggest that acetate may exert protective effects, while changes in propionate and butyrate levels may signal increased cardiovascular risk.

In line with these findings, Hsu et al. [135] examined the roles of SCFA in disease progression in children with early-stage CKD. The study revealed that children with CKD due to congenital anomalies of the kidneys and urinary tract (CAKUT) exhibited lower plasma levels of propionate and were less likely to experience blood pressure abnormalities. In contrast, children with CKD who showed abnormal ambulatory blood pressure profiles had elevated levels of propionate and butyrate.

Additional pediatric studies have investigated the role of gut microbiota in idiopathic nephrotic syndrome, indicating a link between changes in SCFA profiling and dysfunction in regulatory T-cells in these patients [136, 137]. These studies suggested that SCFA may play a role in idiopathic nephrotic syndrome by modulating inflammation.

Wang et al. [138] observed that children who relapsed after glucocorticoid treatment had lower levels of butyrate-producing bacteria at disease onset. Post-treatment microbiota composition improved, but it remains unclear whether post-treatment dysbiosis contributes to relapse risk. These findings highlight the need for longitudinal studies to track microbial changes after treatment and to evaluate whether modulating SCFA levels may reduce relapse frequency.

A recent review by Tain et al. [127] summarized several pediatric studies reporting alterations in gut microbiota composition and metabolite profiles, particularly SCFAs, in children with kidney disorders.

Together, these data suggest that gut-derived SCFAs are relevant biomarkers and potential modulators of pediatric kidney disease progression. Importantly, most of these studies are correlative, and thus, further research is needed to establish causality and identify potential therapeutic targets. Future therapies could explore dietary fiber, probiotics, prebiotics, or even isolated SCFA supplementation to support gut-kidney axis function in children with CKD.

## Therapeutic potential of SCFA signaling modulation

SCFAs may serve both as biomarkers for disease progression and as potential therapeutic agents for the prevention and treatment of pediatric kidney disease (Fig. 4C). Advances in microbial genomics have revealed the enormous metabolic potential of the gut microbiota, especially its ability to produce SCFAs.

Evidence from both lab animals and humans suggests that enhancing SCFA levels may promote vascular health. For example, in different studies, supplementation with *Dendrobium officinale*, a medicinal orchid rich in fermentable fiber, increased both fecal and serum SCFAs, improved endothelial function, and reduced arterial stiffness in rats [139] and humans [140]. These effects appeared to be dependent on endothelial nitric oxide synthase (eNOS), as they were abolished by eNOS inhibition. Notably, only recently have researchers devoted time to understanding specifically how some strains of bacteria produce SCFA more efficiently than others.

Flolova et al. [141] constructed a comprehensive reference collection of SCFA metabolic genes and phenotypes by analyzing 2,856 human gut bacterial genomes from 823 species. Their comparative genomics approach enabled reconstruction of all known pathway variants for butyrate (4 variants), propionate (3 variants), acetate, formate, and lactate. Complete butyrate and propionate synthesis pathways were identified in 359 and 826 genomes, respectively, with many genomes carrying multiple variants. Simpler SCFA pathways, such as those for acetate and formate, were found in 45–87% of genomes. This genomic resource can support metabolic profiling of gut metagenomes, improving the accuracy of in silico predictions for microbiota-based therapeutic studies.

Emerging interventions that leverage SCFA biology include probiotics, prebiotics, dietary fiber, microbiota transplantation, SCFA supplementation, and GPCR-targeted therapies. In a comprehensive review, Magliocca et al. [142] reported that SCFA treatments reduced inflammation and improved kidney function in both in vitro models and animal studies of CKD, suggesting that SCFAs or their bacterial producers could become viable therapeutic agents.

Chang et al. [111], studying the relationship between changes in gut microbiota and hypertension in patients with preeclampsia, found that microbiota diversity and specific SCFA were both reduced in these patients compared to healthy subjects. Moreover, the authors hypothesized that oral administration of butyrate could attenuate LPS-induced hypertension in pregnant rats. Notably, oral supplementation of butyrate significantly decreased the blood pressure in these rats.

Corroborating these results, Jin et al. [107] showed that gut microbiota dysbiosis is common in preeclamptic patients, and these patients have significant reductions in SCFA-producing bacteria and SCFAs. Interestingly, the gut microbiota of preeclamptic patients increased pathologies and symptoms of preeclamptic rats, whereas the gut microbiota of healthy pregnant women had substantial protective effects. Oral treatment with SCFA-producing bacteria, propionate, or butyrate significantly alleviated the symptoms of preeclampsia in rats. The authors found that SCFA treatment promoted autophagy and M2 polarization of macrophages in the placental bed, thereby suppressing inflammation. Propionate also stimulated trophoblast invasion, thereby improving spiral arterial remodeling and placental function.

A recent pilot study by Piteková [143] provided the first pediatric evidence that fecal microbial transplantation can effectively prevent recurrent febrile urinary tract infections by restoring gut microbiota balance and improving metabolome profiles, in particular SCFAs-producing microbes.

Serebrinsky-Duek et al. [144] developed a novel approach, in which synthetic bacterial biosensors can detect the absence of propionate and butyrate using the logic “NOT” gates and specific promoters. These engineered microbes could deliver therapeutic payloads in response to SCFA deficiency, suggesting a programmable therapeutic platform for gut-targeted interventions.

Despite these promising developments, more mechanistic research is needed to clarify how SCFA-mediated pathways influence kidney development, inflammation, and blood pressure regulation. Investigating SCFA-receptor interactions, epigenetic effects, and cell-type-specific signaling, particularly in the developing kidney, may uncover novel therapeutic targets.

Interventions such as dietary fiber supplementation, prebiotics, probiotics, and SCFA analogs could help restore maternal or pediatric microbiota balance, support nephrogenesis, and prevent kidney development defects. As our understanding of the gut-kidney axis deepens, these strategies will offer significant potential for improving outcomes in both pediatric and adult kidney disease.

## Conclusions and future directions

Emerging evidence demonstrates a positive association between elevated levels of colonic and circulating SCFAs and effective blood pressure regulation. In contrast, disruptions in SCFA balance have been linked to metabolic disorders, systemic inflammation, impaired blood pressure control, and progressive kidney dysfunction.

Beyond their intracellular metabolic roles, SCFAs also function as signaling molecules by activating GPCRs. These receptors influence vascular tone, endothelial function, and kidney physiology, processes that are critical for maintaining cardiovascular and kidney health.

Given their multifaceted roles, SCFAs and their receptors have emerged as promising therapeutic targets for the treatment of hypertension and kidney disease. Ongoing and future research will be crucial in translating these findings into clinical interventions. Future directions should include:

- Expanded animal and clinical studies to validate therapeutic efficacy.
- Investigation of tissue- and cell-specific actions of SCFAs and their receptors.
- Exploration of SCFA roles in kidney development.
- Evaluation of SCFA supplementation, prebiotics, and probiotic strategies.
- Assessment of SCFA alterations as diagnostic or prognostic biomarkers.
- Pharmacological studies to develop targeted modulators of SCFA signaling pathways.

By deepening our understanding of SCFA signaling, we open new avenues for early-life interventions and precision therapies aimed at improving long-term cardiovascular and kidney health.

## Supplementary Information

Below is the link to the electronic supplementary material.

Graphical Abstract (PPTX 296 KB)
