# Supplementary material for: Therapeutic strategies for hypertension: exploring the role of microbiota-derived short-chain fatty acids in kidney physiology and development
Source: Pediatr Nephrol. 2025 Jul 10;41(4):937–56. doi: 10.1007/s00467-025-06883-2 (PMC12953496; doi:10.1007/s00467-025-06883-2)
Supplement: Supplementary file 1 — Graphical Abstract (PPTX 296 KB) [file 467_2025_6883_MOESM1_ESM.pptx]

## Slide 1
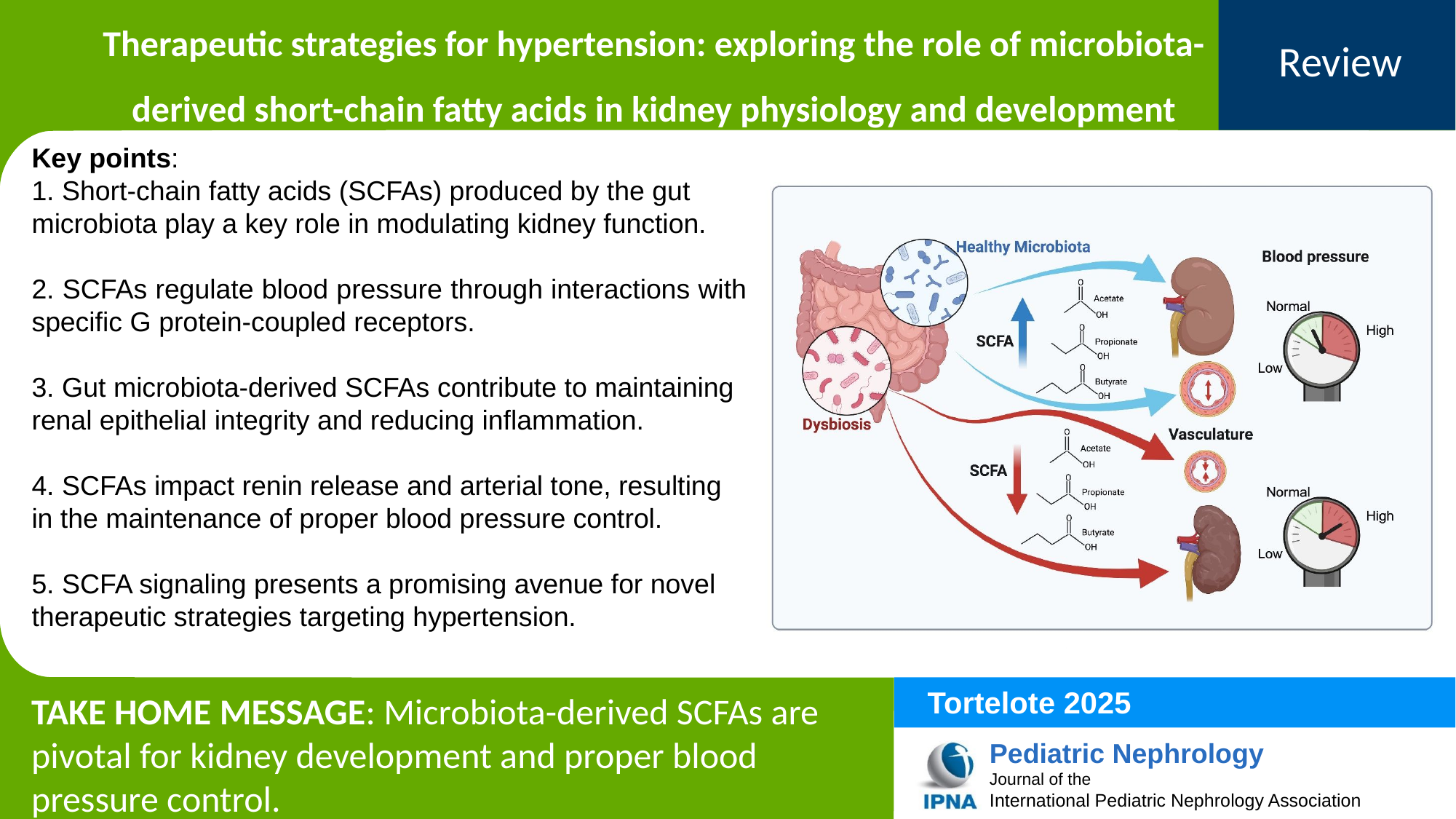

Therapeutic strategies for hypertension: exploring the role of microbiota-derived short-chain fatty acids in kidney physiology and development
Key points:
1. Short-chain fatty acids (SCFAs) produced by the gut microbiota play a key role in modulating kidney function.
2. SCFAs regulate blood pressure through interactions with specific G protein-coupled receptors.
3. Gut microbiota-derived SCFAs contribute to maintaining renal epithelial integrity and reducing inflammation.
4. SCFAs impact renin release and arterial tone, resulting in the maintenance of proper blood pressure control.
5. SCFA signaling presents a promising avenue for novel therapeutic strategies targeting hypertension.
Tortelote 2025
TAKE HOME MESSAGE: Microbiota-derived SCFAs are pivotal for kidney development and proper blood pressure control.
